# Reflected wave intensity increases based on aortic diameter after endovascular aortic therapy in a goat model

**DOI:** 10.1038/s41598-020-80920-y

**Published:** 2021-02-15

**Authors:** Tomohiro Takano, Masumi Iwai-Takano, Yusuke Tsuboko, Yasuyuki Shiraishi, Tomoyuki Yambe, Takashi Igarashi, Hitoshi Yokoyama

**Affiliations:** 1 Department of Cardiovascular Surgery, Fukushima Medical University, 1 Hikarigaoka, Fukushima, 960-1295 Japan; 2 Department of Epidemiology, Fukushima Medical University, Fukushima, Japan; 3 Fukushima Prefectural General Hygiene Institute, Fukushima, Japan; 4 Graduate School of Biomedical Engineering, Tohoku University, Sendai, Japan; 5 Waseda Research Institute for Science and Engineering, Waseda University, Tokyo, Japan; 6 Institute of Development Aging and Cancer, Tohoku University, Sendai, Japan

**Keywords:** Aortic diseases, Arterial stiffening

## Abstract

Reflected wave increases after endovascular aortic repair (EVAR) in patients with aortic aneurysm. This affects the left ventricular (LV) diastolic function and leads to a poor prognosis. This study aimed to evaluate the relationship between increased reflected wave amplitude and aortic diameter after EVAR. EVAR was performed in seven healthy goats. We assessed wave intensity (WI), aortic diameter, and stiffness parameter β. Moreover, we evaluated the relationship between negative reflected wave (NW, reflected waves toward the heart from the periphery by WI) and other parameters after EVAR. Results showed an increase in stiffness parameter β (3.5 ± 0.3 vs 15.9 ± 4.7, *p* = 0.018) and a decrease in the change of aortic diameter (6.9 ± 0.7 vs 2.7 ± 0.4%, *p* = 0.018) after EVAR. The NW was significantly amplified after EVAR from baseline (−589.8 ± 143.4 to  − 1192.3 ± 303.7 mmHg-m/sec^3^, *p* = 0.043). The NW showed a significant correlation with maximum aortic diameter (R = 0.707, *p* = 0.038) and minimum aortic diameter (R = 0.724, *p* = 0.033). The reflected wave was enhanced after EVAR and was correlated to the aortic diameter at the stent-graft site. It is important to consider that patients with smaller aortic diameters in landing zone who undergo EVAR may develop LV dysfunction.

## Introduction

The minimally invasive nature of endovascular aortic repair (EVAR) has led to a rapid increase in its usage in the last two decades. EVAR is recommended for elderly patients with aortic aneurysm as a substitute for conventional aortic grafting. However, it has been reported that cardiovascular events and deaths increase during the long-term follow-up period after EVAR^1,2^. A greater ascending aorta diameter, presence of mitral regurgitation, reduced left ventricular (LV) ejection fraction, younger age, diabetes mellitus, and the female sex were independently associated with long-term mortality after EVAR^2,3^. Furthermore, it has been reported that increased aortic vascular stiffness (pulse wave velocity: PWV), LV hypertrophy, LV diastolic dysfunction, and exercise intolerance were observed 1 year after EVAR^4^.

To clarify the mechanism of hemodynamic change after EVAR, several investigators studied the change in hemodynamics and/or vascular characteristics. Reports showed that EVAR caused an increase in afterload and LV work and resulted in cardiac hypertrophy during the follow-up period^4,5^.

On the other hand, it has also been reported that reflected wave increased after EVAR^6^, and caused LV diastolic dysfunction^7,8^. Thus, it is important to clarify which factors enhanced the reflected wave after EVAR.

PWV correlates with vascular diameter^9,10^, and an increased PWV leads to an early reflected wave^7,11^. However, it remains unclear whether the aortic diameter, where the stent-graft is deployed, is related to the enhancement of the reflected wave after EVAR.

Thus, the purpose of this study was to evaluate the relationship between the aortic diameter and an increased reflected wave after EVAR in vivo.

## Results

We studied seven goats (four females, body weight 61.4 ± 5.4 kg). EVAR was performed with a stent-graft size (diameter of 23.1 ± 0.6 mm, length of 97.1 ± 8.6 mm) according to each aortic diameter.

### Hemodynamic parameters

The changes in hemodynamic parameters from baseline to after EVAR are shown in Table 1. There were no significant changes in heart rate, cardiac output, systolic and diastolic aortic pressures, systolic and diastolic pulmonary pressures, LV ± dP/dt, LV ± dQ/dt and Z0 as aortic input impedance after EVAR from baseline. Moreover, there were no significant differences between baseline and after EVAR in forward pressure (Pf) and backward pressure (Pb), reflection magnitude (RM) and reflection index (RI). On the other hand, there was a significant increase in pulse pressure (23.6 ± 1.8 to 29.4 ± 2.9 mmHg, *p* = 0.046) and Zc as characteristic impedance (62.8 ± 4.9 to 85.2 ± 6.9 dyn-sec/cm^5^, *p* = 0.018) after EVAR.

**Table 1:** Hemodynamic parameters at baseline and after stent-grafting.

	Baseline	After EVAR	*p* value
Body weight (kg)	61.4 ± 5.4		
Stent-graft size			
Diameter (mm)	23.1 ± 0.6	–	
Length (mm)	97.1 ± 8.6	–	
Heart rate (bpm)	119.6 ± 5.9	119.9 ± 5.6	0.889
Cardiac output (l/min)	4.7 ± 0.3	4.6 ± 0.2	0.866
Aortic pressure			
Systole (mmHg)	114.0 ± 6.5	114.3 ± 6.0	0.753
Diastole (mmHg)	90.4 ± 5.1	84.9 ± 5.0	0.310
Mean (mmHg)	98.0 ± 5.5	94.7 ± 5.2	0.672
Pulse pressure (mmHg)	23.6 ± 1.8	29.4 ± 2.9	0.046
Pulmonary pressure			
Systole (mmHg)	22.0 ± 2.1	23.6 ± 3.0	0.168
Diastole (mmHg)	8.7 ± 2.4	10.6 ± 2.6	0.058
Mean (mmHg)	13.1 ± 2.3	15.1 ± 2.5	0.042
LV + dP/dt (mmHg/sec)	2886.7 ± 257.9	2898.2 ± 223.0	0.917
LV −dP/dt (mmHg/sec)	−1770.0 ± 271.4	−1796.5 ± 388.0	0.753
LV + dQ/dt (ml/sec)	812.3 ± 73.5	872.8 ± 99.3	0.753
LV −dQ/dt (ml/sec)	−472.3 ± 62.2	−392.2 ± 72.8	0.116
Z0 (dyn sec cm^−5^)	1757.0 ± 62.9	1780.0 ± 158.3	0.735
Zc (dyn sec cm^−5^)	62.8 ± 4.9	85.2 ± 6.9	0.018
Forward pressure (mmHg)	108.4 ± 5.9	108.1 ± 5.7	1.0
Reflected pressure (mmHg)	7.8 ± 0.6	9.4 ± 0.9	0.128
Reflection magnitude	0.46 ± 0.2	0.43 ± 0.33	0.237
Reflection index	0.32 ± 0.00	0.30 ± 0.17	0.128

*EVAR* endovascular aortic repair, *LV* left ventricle, Value, mean ± SE.

### Change in aortic diameters, stiffness parameter β and local PWV

There was no significant difference in the maximum and the minimum aortic diameters at the stent-graft site before (baseline) and after EVAR. However, the change in aortic diameter significantly decreased after EVAR (baseline vs after EVAR; 6.9 ± 0.7 vs 2.7 ± 0.4%, *p* = 0.018).

There were significant increases in the stiffness parameter β (3.5 ± 0.3 vs 15.9 ± 4.7, *p* = 0.018) and local PWV (4.3 ± 0.4 vs 8.0 ± 0.9 m/sec, *p* = 0.018) after EVAR compared to those at baseline (Table 2).

**Table 2:** Change in aortic diameters, stiffness parameter beta, local PWV and wave intensity at baseline and after stent-graft.

	Baseline	After EVAR	*p* value
**Aortic diameter**			
Maximum (mm)	19.7 ± 1.7	19.3 ± 1.6	0.612
Minimum (mm)	18.4 ± 1.7	18.8 ± 1.5	0.612
Change of diameter (%)	6.9 ± 0.7	2.7 ± 0.4	0.018
**Stiffness parameter beta**	3.5 ± 0.3	15.9 ± 4.7	0.018
**Local PWV (m/s)**	4.3 ± 0.4	8.0 ± 0.9	0.018
**Wave intensity (mmHg.m/s**^**3**^**)**			
Wave 1	9693.9 ± 454.7	16,201.3 ± 2480.2	0.018
Wave 2	5708.5 ± 1520.3	4308.3 ± 988.2	0.612
Negative wave	−589.8 ± 143.4	−1192.3 ± 303.7	0.043

Value, mean ± SE; *PWV* pulse wave velocity by Bramwell–Hill equation.

### Wave intensity (WI)

There was a significant increase in W1 (first peak) after EVAR (baseline vs after EVAR; 9693.9 ± 454.7 vs 16,201.3 ± 2480.2 mmHg-m/sec^3^, *p* = 0.018), but not in the W2 (second peak). In contrast, the NW (negative reflected wave) was significantly enhanced after EVAR compared to baseline measurements (baseline vs after EVAR;  − 589.8 ± 143.4 vs  − 1192.3 ± 303.7 mmHg-m/sec^3^, *p* = 0.043) (Table 2).

### Correlation with negative wave

We assessed which parameter correlated with the NW. For baseline measurements, there was no correlation between NW and any parameters.

**Figure 1:**
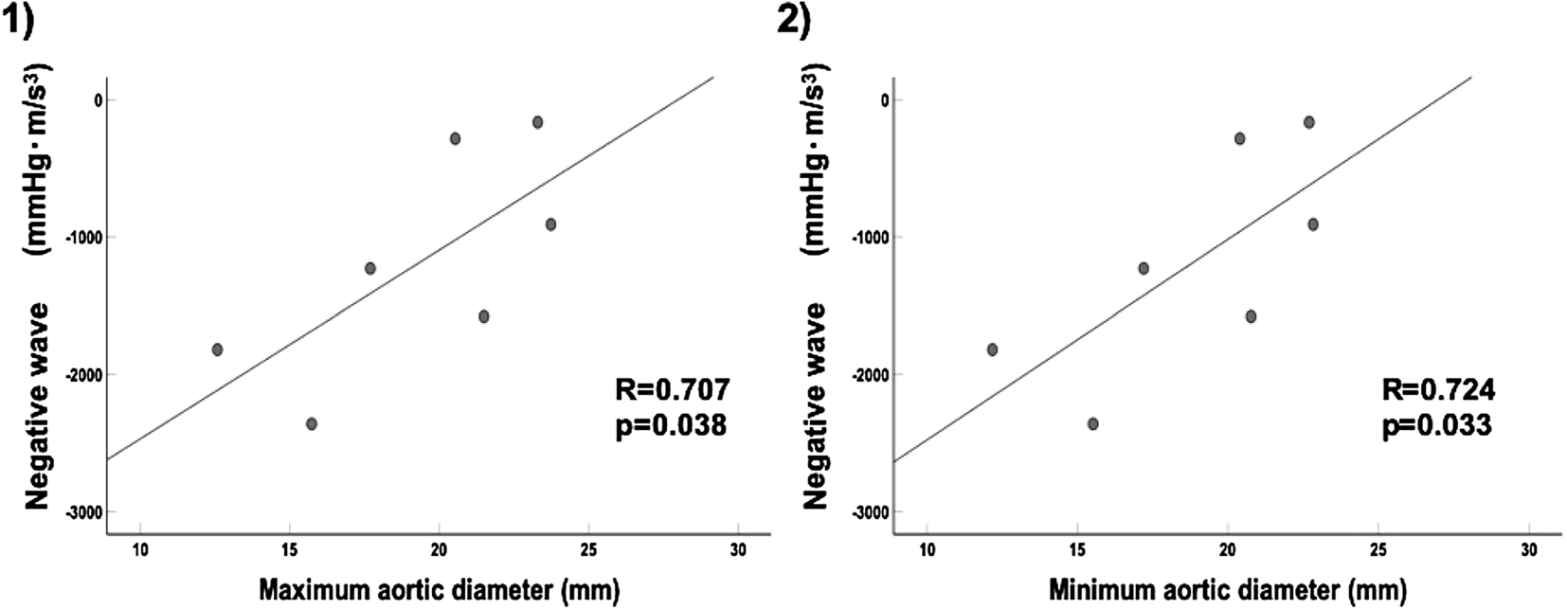
Correlation with negative wave and aortic diameters after EVAR. The NW had significant correlations with maximum aortic diameter (**1**) and minimum aortic diameter (**2**). The greater absolute values of NW show the greater of NW intensity.

**Figure 2:**
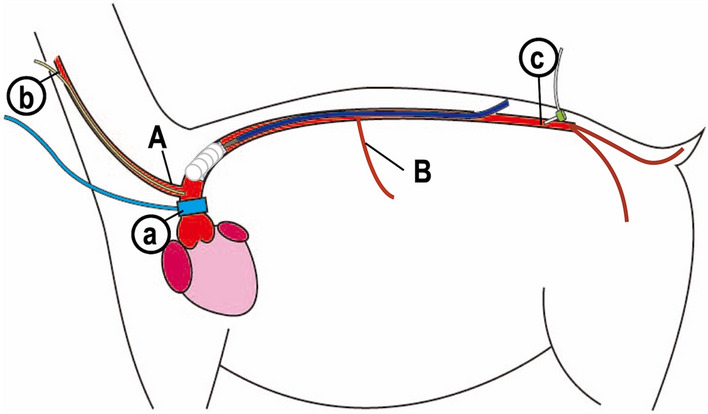
A schematic overview of animal preparation, stent-grafting, and study protocol. A goat was placed in the right decubitus position. A flowmeter probe attached to the aortic root (a), jugular arterial line (b), and terminal arterial line (c) was introduced. Stent-graft was deployed between the distal end of the cervical trunk (A) and the celiac trunk (B).

After EVAR, there was no correlation between NW and any hemodynamic parameters. Among the parameters of aortic stiffness, the NW showed a significant correlation with maximum aortic diameter (R = 0.707, *p* = 0.038, Fig. 1-1) and minimum aortic diameter (R = 0.724, *p* = 0.033, Fig. 1-2) (Table 3). However, the NW did not correlate with the length of stent-graft. Moreover, there were no relationship between the change in NW and the change in hemodynamic parameters or stiffness indices.

**Table 3:** Relationship between negative wave intensity and the parameters of aortic stiffness after EVAR using univariate analysis.

	R	*p* value
Zc	0.386	0.196
Maximum aortic diameter	0.707	0.038
Minimum aortic diameter	0.724	0.033
Change of aortic diameter	−0.135	0.387
Stiffness parameter beta	0.204	0.331
Local PWV	−0.063	0.447

## Discussion

Our major findings were as follows (1) Negative wave intensity, which is indicated by backward waves reflected toward the heart from the periphery, was significantly enhanced after EVAR compared to that at baseline; and (2) Negative wave intensity correlated with the aortic diameters at stent-graft site. To the best of our knowledge, this is the first study to identify that the reflected wave after EVAR correlated with aortic diameter in vivo.

LV diastolic dysfunction is related to reflected waves, including cardio-arterial interaction, in patients after EVAR^3,4^. In general, the reflected waves from peripheral vessels reach the ascending aorta in early diastole. However, when the elastic properties of the aortic wall are diminished by EVAR, the reflected wave returns quickly to the ascending aorta, and it is fused to the systolic phase^12^. Alderson et al.^13^ reported that the placement of a rigid stent within an elastic vessel produces wave reflection sites at the entrance to and exit from the stent^13^. This phenomenon is produced by impedance mismatch which is generated as a stepwise transition from elastic to rigid boundary conditions as the flow enters the rigid segment and the reverse when it exits. Thus, the main reflection site is moved from the terminal aorta to the entrance and exit of the stent-graft after EVAR, and the reflected wave is enhanced.

PWV, defined as the speed with which the pulse travels in the aorta (distance/time), is directly related to the elastic properties of the aortic wall^12^. Tzilalis et al.^12^ reported that PWV was greater in younger patients who underwent EVAR for thoracic aortic injuries or aortic dissections compared to the healthy control group^12^. In addition to that, Beaufort et al.^14^ described that the increase in PWV showed a positive linear correlation with the percentage of total aortic length covered by the stent-graft, and assumed that a longer range of the aorta becomes more rigid, thus decreased aortic elasticity, which led to the increase in PWV^14^. PWV is defined as Moens-Korteweg equation $\left( {{\text{PWV}} = \sqrt {\left( {{\text{Eh}}/{2}\rho {\text{r}}} \right)} } \right);$ E: elastic modulus, h: wall thickness, ρ: blood density, r: vessel radius)^15^. Thus, the increased elastic modulus leads to a greater PWV and the reflected wave is enhanced after EVAR.

In this study, stiffness parameter β and local PWV, which are indexes of arterial stiffness, were increased. This finding in the healthy aorta without arteriosclerosis was in line with that in the patients with arteriosclerosis after EVAR^4,5^. On the other hand, some investigators reported that there was no relationship between PWV and augmentation index^6^. Augmentation index integrates stiffness, wave reflection and cardiac function same as negative wave intensity. These are according with the result of our study.

In addition, we showed a significant correlation between negative wave intensity and aortic diameter in healthy goat models. From the Means–Korteweg equation, PWV was greater when the aortic diameter (r) was smaller^15^. Hacham et al.^16^ also reported that reflected wave intensity showed a negative correlation with the diameter in an elastic tube of stenosis model^16^. Thus, our findings suggest that the assessment of the aortic diameter is important for patients who undergo EVAR. When EVAR is performed in patients with small aortic diameters, the intensity of reflected waves increases, and this might lead to a poor prognosis according to LV diastolic dysfunction. Therefore, further research is required to confirm the relationship between the aortic diameter and prognosis of patients with aneurysm after EVAR.

In our study, W1 was significantly increased after EVAR. The W1 occurs during early systole, the magnitude of which increases with increases in cardiac contractility^17^. Jones et al.^18^ clarified that administration of dobutamine increased W1 in dogs and there was a significant difference between W1 and max dP/dt^18^. In this study, W1 was increased after EVAR. However, no correlations between W1 and the other hemodynamic parameters were observed. Thus, the mechanism of enhanced W1 after EVAR remains unclear. An acute change in the arterial stiffness might be influenced by an increase of W1.

EVAR is established as a less-invasive procedure that is effective for frail elders, specifically; however, it should be used with caution due to the change in cardiac properties produced by the expansion of the reflected wave. In particular, our data suggest that the reflected wave may be significantly affected after EVAR in patients who have a smaller aortic diameter in the landing zone, which is the case in elderly women. Thus, the change in cardiac function should be assessed, i.e., LV diastolic function or exercise tolerability, over a long-term follow-up period. Moreover, a stent-graft with increased flexibility should be developed that would preserve vascular function and prevent the enhancement of the reflected wave.

This study had several limitations. First, this was an experimental study using healthy goats, and not atherosclerotic nor aneurysmal models. Thus, the hemodynamic effect after EVAR may be underestimated because healthy aortas may compensate for the adverse effect of EVAR. A further study of the atherosclerotic model would be required. Secondly, we used a small number of goats. Therefore, our findings will need to be confirmed in studies with a larger sample size. Thirdly, this study focused on the acute change in hemodynamics after EVAR. It is possible that we may not have detected the impact of reflected wave on the heart in this study. Moreover, it remains unclear whether our findings are observed in the chronic phase. Finally, EVAR was performed in the thoracic aorta, not the abdominal aorta, in our study. It is necessary to investigate whether the effect of the position of EVAR is different between the thoracic and abdominal aortas in the future.

Our study revealed that the reflected wave toward the heart from the periphery is significantly enhanced after EVAR, and the reflected wave is associated with the aortic diameter of stent-graft site. This highlights the importance of considering the possibility of LV dysfunction developing in patients with a smaller aortic diameter in landing zone, who undergo EVAR with small stent-graft. Thus, there is a need to carefully determine diastolic dysfunction during follow-up periods.

## Methods

### Animal preparation

After fasting for 24 h, the goats (n = 7, four females, body weight 61.4 ± 5.4 kg) were anesthetized through inhalation of 5% isoflurane in the right lateral decubitus position and tranquillized with an intravenous injection of vecuronium bromide (0.5–1.0 mg/kg) and atropine sulfate hydrate (1 mg), and was maintained with 1.5–2% isoflurane. The heart rate, blood pressure, and blood O_2_ saturation were constantly monitored using an anesthetic apparatus (Vp-1000; IMI, Saitama, Japan) and polygraph system (MCS-9000; Fukuda Denshi, Tokyo). Venous access was established for periprocedural hydration and drug administration through the jugular vein. In addition, we inserted 5Fr catheter from the common carotid artery with a single pressure transducer (Meritrans DTXPlus; Merit Medical Japan, Tokyo) and Swan-Ganz catheter from the jugular vein. After the heart was exposed via thoracotomy, an ultrasonic flowmeter probe was attached to the aortic root, and a velocity transducer was energized with a 40-Hz frequency response (PAX; Transonic Systems. Inc, NY, USA). A Millar microtip catheter (Millar, Texas, USA) was also inserted via the terminal aorta to the LV (Fig. 2).

We performed EVAR according to standard clinical procedure and monitored hemodynamic parameters at baseline and after EVAR. Any inotropic or vasoactive agents were not administered during all procedures.

This study was approved by the Institutional Laboratory Animal Care and Use Committee of Tohoku University (2016AcA-034, 2017AcA-053) and was performed according to the guidelines for the proper conduct of animal experiments by the Science Council of Japan.

### Stent-grafting

After the abdominal aorta was exposed, a heparin (1000 U/kg) bolus was injected. We clamped the abdominal aorta. Then, a TX-2 thoracic stent-graft with a diameter of 22–26 mm and a length of 80–135 mm (COOK Medical LLC, Bloomington, USA) was introduced over the wire through the abdominal aorta directly and was advanced to the descending thoracic aorta. The stent-graft was deployed between the distal end of the cervical trunk and the celiac trunk under ultrasound guidance (Fig. 2). We selected the stent-graft size, which was oversized by between 10 and 25% of each aortic diameter. Post-ballooning was not performed.

### Hemodynamic parameters

We evaluated the hemodynamic parameters including heart rate (bpm), LV pressures (mmHg) using Millar catheter, cardiac output (L/min) using flowmeter, systolic and diastolic aortic pressures (mmHg), systolic and diastolic pulmonary arterial pressures (mmHg) using the fluid-filled method. All digitized data of the parameters were subsequently analyzed with Mathematica (Wolfram Research, Champaign, IL, USA). To maintain fluctuations in pressure and velocity signals caused by uncontrollable movement of the catheter, aortic pressure and velocity were ensemble-averaged over eight beats using the peak of the R wave of the electrocardiogram to indicate the beginning of the beat.

**Figure 3:**
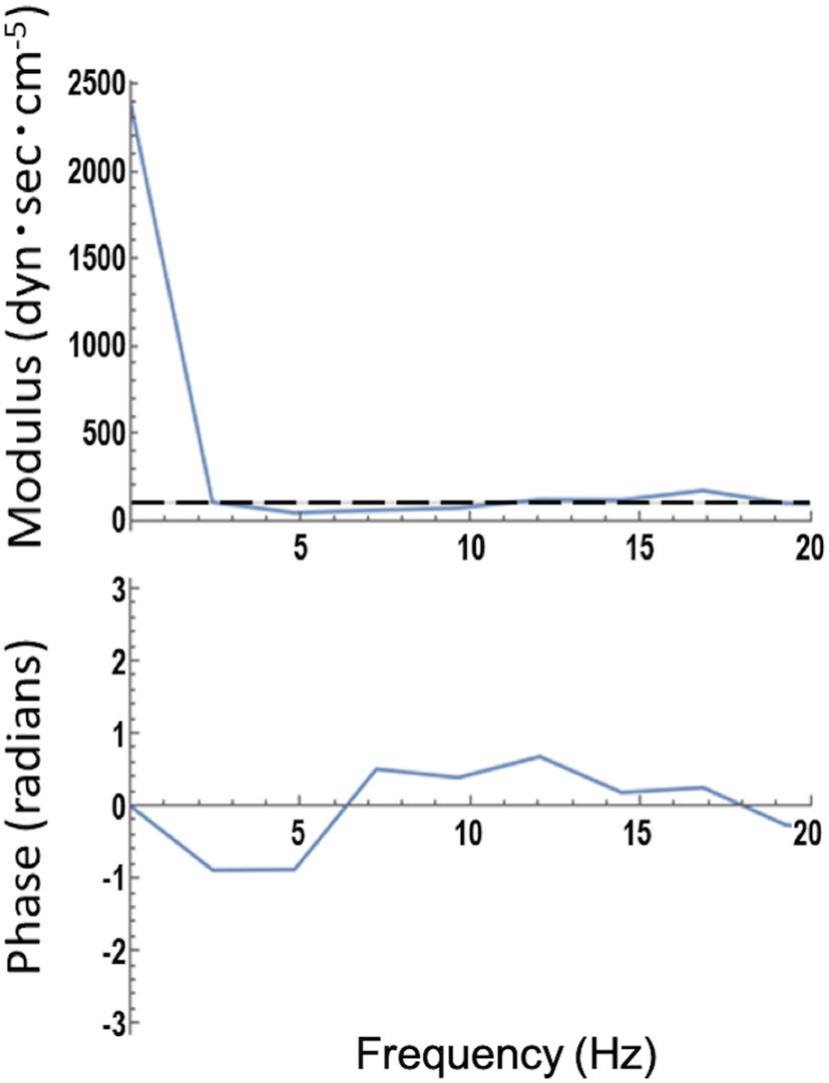
Representative example of aortic input impedance modulus and phase. Dashed line shows characteristic impedance.

We assessed Z0 and Zc. Z0, which is calculated from the Fourier transform of the ascending aortic pressure and flow waveforms, represents LV afterload^19^. Zc is the ratio of pulsatile pressure to pulsatile flow in the absence of wave reflections and is attributed to proximal aortic wall stiffness and radius^20^. It was calculated as the average value of the input impedance over the frequency range of 1 to 10 Hz (Fig. 3).

We also evaluated forward pressure (Pf) and backward pressure (Pb), RM and RI. The RM is calculated as the ratio of the amplitudes (peak-trough) of the backward and forward pressure waves: RM =|Pb|/|Pf|. The RI is defined as: RI = Pb/(|Pf| +|Pb|)^21^.

### Wave intensity

WI is a hemodynamic index that provides information on the dynamic behavior of the heart, the vascular system, and their interactions. WI was defined as WI = dPdU, i.e., the product of dP and dU, where dP and dU are the changes in blood pressure (P) and velocity (U) during constant short time intervals^17^. Actually, we used 1000 Hz sample rate, and calculated dP and dU from the crude sample-to-sample differences per 0.001 s.

**Figure 4:**
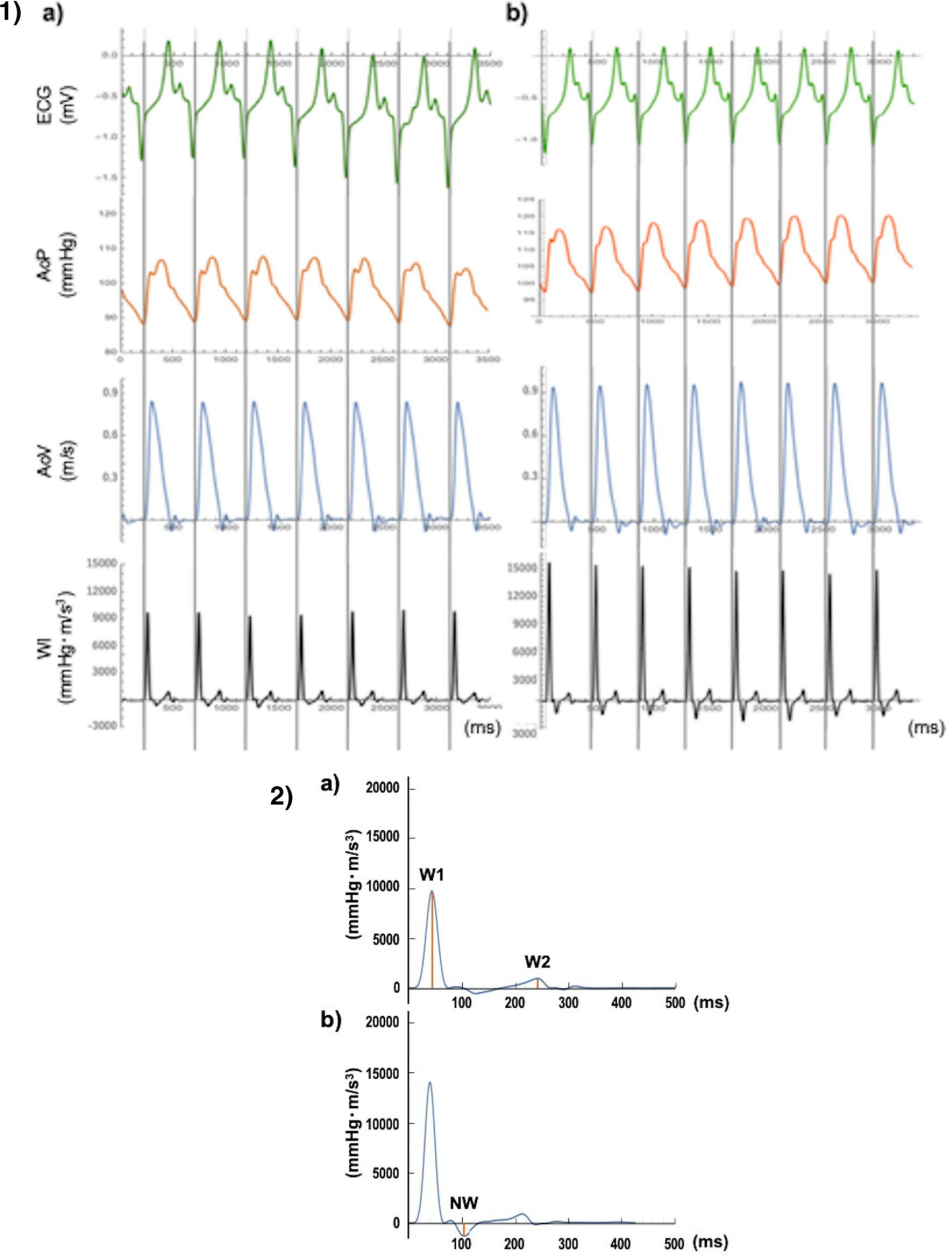
(**1**) Representative examples of waveforms measured at baseline (a) and after EVAR (b). ECG, ascending aortic pressure (AoP), aortic velocity (AoV) and calculated wave intensity (WI) obtained from eight successive beats in the ascending aorta. (**2**) Representative case of wave intensity at baseline (a) and after EVAR (b). There was an increase in W1 and a decrease in NW after EVAR. W1: first peak during early systole, W2: second peak during end of ejection, NW: backward wave in mid systole.

WI was calculated by using eight consecutive waveforms of pressure and flow. The peaks of W1, W2 and negative wave were extracted from the average for eight waveforms by using Mathematica (Fig. 4-1,-2).

WI is divided into three major parts, two positive waves, and a negative wave. W1 occurs during early systole, where the magnitude increases with an increase in cardiac contractility. W2, which occurs towards the end of ejection, is related to the ability of the left ventricle to actively stop aortic blood flow^17^. During mid-systole, NW represents backward waves reflected toward the heart from the periphery^22^. We assessed the magnitude of W1, W2, and NW at baseline and after EVAR.

### Aortic diameter

**Figure 5:**
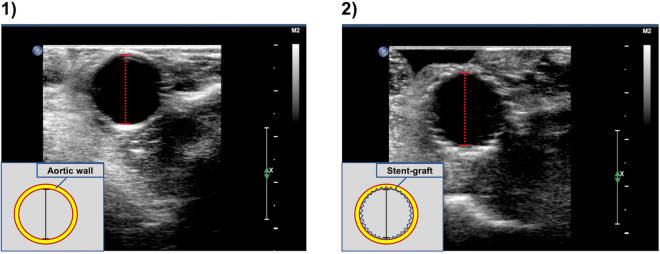
Ultrasound imaging of a cross section of the aorta at baseline (**1**) and after EVAR (**2**). The internal aortic diameters at the stent-graft site were measured as aortic diameter at baseline and after EVAR.

Aortic diameter, in which the stent-graft was implanted, was evaluated by B-mode ultrasonography (iE33 with S5-1 probe; Philips, Bothell, WA, USA) at baseline (before) and after EVAR. The internal aortic diameters were measured as maximum aortic diameter (Dmax) and minimum aortic diameter (Dmin) (Fig. 5). Then, we calculated the change in aortic diameter as;

Change of aortic diameter = (Dmax − Dmin)/Dmin.

### Stiffness parameter β

Stiffness parameter β is the slope of the exponential function between the relative arterial pressure and the distention ratio of artery, and it is given as^23^:

Stiffness parameter β = ln(SBP/DBP)/[(Dmax − Dmin)/Dmin].

(SBP: systolic blood pressure, DBP: diastolic blood pressure).

In this study, ascending aortic pressures (SBP and DBP) during ultrasonography were substituted for pressures at stent-graft site.

### Local PWV

PWV is a representative parameter of arterial stiffness. Local wave speed was calculated by using the Bramwell–Hill’s equation^9^^,^^24^, which is as follows:

$${\text{PWV }} = \, \sqrt {\left( {{\text{Ad}}*{\text{PP}}/\rho *\Delta {\text{A}}} \right)}$$

Ad: cross-sectional area at diastole, ΔA: the difference in cross-sectional area between systole and diastole, PP: local pulse pressure, ρ: blood density (1060 kg/m^3^).

In this study, ascending aortic pulse pressures during ultrasonography were substituted for pressures at the stent-graft site.

### Statistical analysis

Statistical analyses were performed using SPSS version 26 (IBM, New York, USA). All continuous variables are expressed as means ± standard error (SE). The comparison of parameters between baseline and after EVAR was assessed using the Wilcoxon **s**igned-rank test. The relationship between the negative wave intensity and the other parameters was evaluated using Pearson’s rank correlation test. A *P* value of < 0.05 was considered statistically significant.
